# Simultaneous Determination of Uric Acid and Xanthine Using a Poly(Methylene Blue) and Electrochemically Reduced Graphene Oxide Composite Film Modified Electrode

**DOI:** 10.1155/2014/984314

**Published:** 2014-11-11

**Authors:** Gen Liu, Wei Ma, Yan Luo, Deng-ming Sun, Shuang Shao

**Affiliations:** Department of Chemistry and Materials Science, Huaibei Normal University, Huaibei 235000, China

## Abstract

Poly(methylene blue) and electrochemically reduced graphene oxide composite film modified electrode (PMB-ERGO/GCE) was successfully fabricated by electropolymerization and was used for simultaneous determination of uric acid (UA) and xanthine (Xa). Based on the excellent electrocatalytic activity of PMB-ERGO/GCE, the electrochemical behaviors of UA and Xa were studied by cyclic voltammetry (CV) and square wave voltammetry (SWV). Two anodic sensitive peaks at 0.630 V (versus Ag/AgCl) for UA and 1.006 V (versus Ag/AgCl) for Xa were given by CV in pH 3.0 phosphate buffer. The calibration curves for UA and Xa were obtained in the range of 8.00 × 10^−8^~4.00 × 10^−4^ M and 1.00 × 10^−7^~4.00 × 10^−4^ M, respectively, by SWV. The detection limits for UA and Xa were 3.00 × 10^−8^ M and 5.00 × 10^−8^ M, respectively. Finally, the proposed method was applied to simultaneously determine UA and Xa in human urine with good selectivity and high sensitivity.

## 1. Introduction

Graphene oxide (GO) and graphene are promising materials with many interesting properties for applications [1]; for example, they have nice compatibility with biological materials such as amino acids [2] and proteins [3]. Although its badly electrical conductivity limits the usage of graphene oxide, many efforts have improved the methods to obtain the reduced graphene oxide, such as chemical reduced [4, 5], UV-induced photocatalytic reduced [6], and electrochemical reduced [7]. Electrochemically reduced graphene oxide could be prepared based on the research of Zhou et al. [8]. Moreover, Casero et al. immersed a graphite working electrode in GO dispersion and controlled an appropriate cathodic potential in 12 hours to obtain reduced graphene oxide [9].

The electron pair or electron cloud overlapping between dye molecules and graphite electrode strengthened their adsorption affinity and accelerated electron transfer rate [10]. Because of the *π*-*π* noncovalent interaction, some electroactive water-soluble aromatic dyes could absorb on the surface of carbon material, such as the combination of Toluidine blue O and multiwall carbon nanotube [11]. Methylene blue (MB), one of the phenoxazine biological dyes, is an electron carrier activation. Sun et al. had applied poly(methylene blue) functionalized graphene modified carbon ionic liquid electrode to determine dopamine. The modified electrode exhibited better electrochemical performances with higher conductivity and lower electron transfer resistance [12]. It indicates that poly(methylene blue) functionalized graphene possesses distinct properties in electrochemical field.

Uric acid (UA, C_5_H_4_N_4_O_3_) is one of the final metabolites of proteins and nucleic acids. It is mainly excreted by kidney and to a less extent by liver. To most mammals and birds, UA is biologically decomposed to allantoin by the action of uricase and then decomposed to NH_3_, CO_2_, and H_2_O. However, the further step is not applicable in humans and apes because of uricase insufficiency. Consequently, UA is the final metabolic product of purine. The amount of UA in human body has great clinical values since it has a bearing in the diagnosis of gout, Lesch-Nyhan syndrome, urolithiasis, kidney damage, leukemia, lymphoma [13–15], and so forth.

Xanthine (Xa, C_5_H_4_N_4_O_2_) mainly exists in the animals' blood, liver, and urine and is an important metabolic intermediate of the purine nucleotide and deoxynucleotide. In addition, the decomposition of adenosine triphosphate (ATP) can also produce Xa [16]. In clinical diagnosis, Xa usually acts as a sensitive indicator for some clinical disorders such as perinatal asphyxia and adult respiratory distress syndrome [17–19]. As the metabolic precursor of UA, Xa determines the level of UA in body fluid. Thus, developing a stable, sensitive, and selective sensor for UA and Xa has considerable importance for clinical analysis. Previously, various methods for the determination of UA and Xa have been reported, such as fluorimetry [20, 21], spectrophotometry [22, 23], enzyme assay [15, 24], chromatography [25, 26], and electrochemical methods [27–29]. However, some inherent problems limit their applications, which includes expensive instruments, complex operations, and personnel training.

In this work, we fabricated poly(methylene blue) and electrochemically reduced graphene oxide composite film modified electrode, which comprised not only excellent electrocatalytic activity for the oxidation of UA and Xa, but also good sensitivity, wide linearity, high selectivity, and remarkable reproducibility. Thus, this modified electrode was satisfactorily used for the simultaneous determination of UA and Xa in human urine by square wave voltammetry (SWV).

## 2. Experimental

### 2.1. Instruments and Chemicals

Electrochemical measurements were performed with a BAS 100 B/W electrochemical workstation (BAS, USA). A conventional three-electrode system was used for all electrochemical experiments, which consisted of an Ag/AgCl (saturated KCl) as the reference electrode, a platinum wire as the auxiliary electrode, and a bare GCE (3 mm in radius) or modified GCE as working electrode. The morphology of the samples was observed using scanning electron microscopes (SEM) from JSM-6610LV (JEOL, Japan). The UV-visible spectra were obtained by UV-3600 UV-VIS-NIR spectrophotometer (Shimadzu, Japan). All pH measurements were performed using a PHS-3C digital pH meter (Shanghai Leici Device Works, China).

Graphene oxide dispersion (2 mg*·*mL^−1^; dispersing agent: water; radius: 1~5 *μ*m; thickness: 0.8~1.2 nm; single layer ratio: 99%; purity: 99%) was obtained from Nanjing XFNANO Materials Technology Company, China. MB, UA, and Xa were purchased from Sigma-Aldrich.All other chemicals were used of analytical grade. Double distilled water was used throughout the experiments. The pH of phosphate buffered solution (PBS) was adjusted with 0.1 M H_3_PO_4_ and 0.1 M NaOH. All experiments were carried out at room temperature.

### 2.2. Preparation of PMB-ERGO/GCE

The bare GCE was polished with 0.05 *μ*m *α*-alumina powder, until a mirror-like finish, rinsed with 1 : 1 HNO_3_ solution, ethanol, and doubly distilled water in an ultrasonic aqueous bath successively, and again rinsed with water before it was used. 1.00 mg*·*mL^−1^ GO in 0.1 M PBS (pH 6.0) was ultrasonicated for 5 min; then an appropriate amount of MB was added to form an aqueous solution (2.00 × 10^−3^ M), followed by ultrasonication for another 5 min. Finally, the polymeric film was electropolymerized by sweeping from 0.7 to −1.2 V at a scan rate of 0.10 V*·*s^−1^ for 11 cycles using cyclic voltammetry. After polymerization, the modified electrode was removed, rinsed thoroughly with double distilled water, and air-dried. Finally the poly(methylene blue) and electrochemically reduced graphene oxide composite film modified electrode (PMB-ERGO/GCE) was successfully prepared.

## 3. Results and Discussion

### 3.1. Characterizations of PMB-ERGO/GCE

Compared with lower and higher electropolymerized cathodic potential, potential of −1.2 V was enough to guarantee good catalytic ability of PMB-ERGO/GCE toward UA and Xa. Besides, lower cathodic potential usually produced hydrogen bubbles on the surface of electrodes without further improving the responses to UA and Xa. In fact, these bubbles not only influenced the electric current density, but also damaged the modified film.

**Figure 1:**
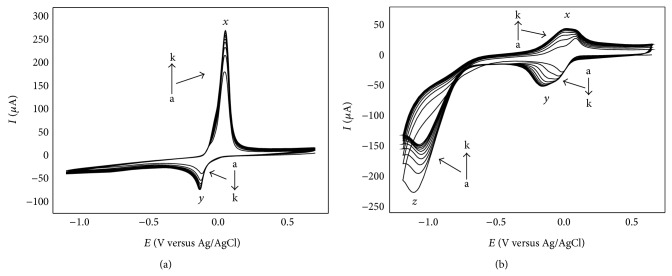
CVs of PMB/GCE (a) and PMB-ERGO/GCE (b) in the polymerization process at a scan rate of 0.10 V*·*s^−1^ for 11 cycles (from a to k); *x*: anodic peak of MB; *y*: cathodic peak of MB; *z*: cathodic peak of GO.

Figure 1(a) gives the cyclic voltammograms of electrochemical polymerization for poly(methylene blue) film modified electrode (PMB/GCE), and Figure 1(b) is PMB-ERGO/GCE. It can be seen in Figure 1(a) that a pair of sharp redox peaks of MB appear at 0.162 V (anodic peak) and −0.181 V (cathodic peak) in the first cycle. This redox reaction possesses poor reversibility in that a large and obvious difference exists in the redox peak currents. In Figure 1(b), however, the anodic peak (at 0.151 V) and the cathodic peak (at 0.022 V) of MB are both wide; moreover, GO has an irreversible cathodic peak at −1.079 V. In the following cycles, the cathodic peak current of GO decreases, the redox peak currents of MB increase, and finally these curves are not changed because of the increasing thickness of modified film and the deceleration of the rate of deposition.

It is well-known that some oxygen-containing functional groups, such as –OH, –COOH, –C=O, –C–O–C–, which exist in graphene oxide, can lead to the large amount of* sp* ^*3*^-hybridized carbon atoms. Electrochemical reduction is a technique to remove these oxygen functional groups and can restore* sp* ^*2*^-hybridized carbon atoms, which can change graphene oxide to graphene. After polymerization, the electrochemically reduced graphene oxide (ERGO) is prepared on the surface of electrodes.

**Figure 2:**
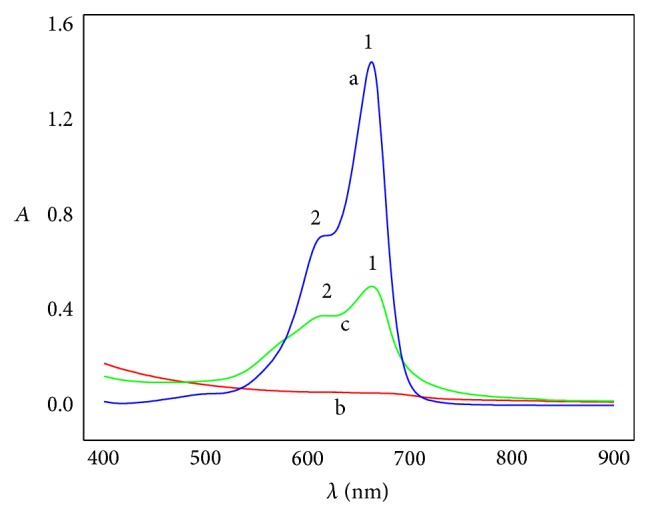
Comparison of UV-visible spectra of MB (a), GO (b), and the mixture solution of MB and GO (c) in 1.0 cm light path length cell.

Figure 2 exhibits UV-visible spectra curves of 1.50 × 10^−5^ M MB and 0.02 mg*·*mL^−1^ GO and the mixture solution of 1.50 × 10^−5^ M MB and 0.02 mg*·*mL^−1^ GO. MB gives two absorption peaks at 662 nm (1) and 616 nm (2), and peak 1 is much spikier and stronger than peak 2. The absorption peaks might originate from the corresponding monomer and dimer of MB, respectively [35]. GO has no obvious absorption. After GO is added to MB solution, the absorption peaks of MB decline drastically, but still at the same wavelength, implying that some interactions occur between MB and GO.

**Figure 3:**
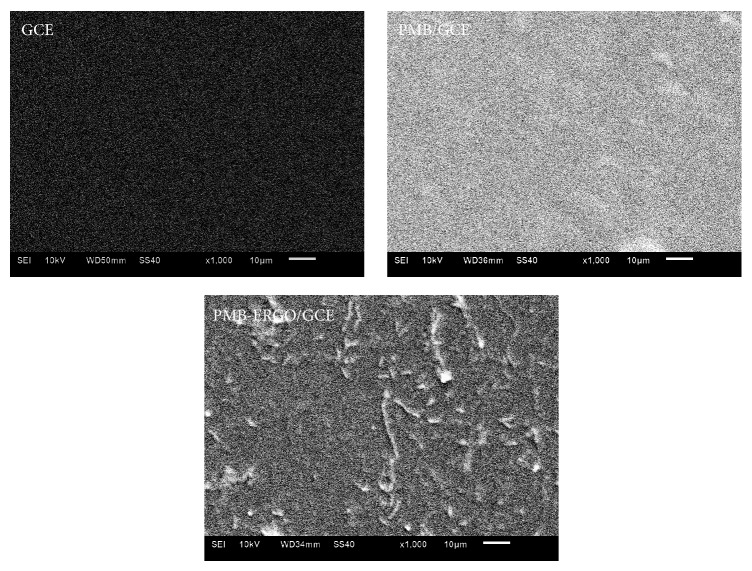
SEM images of GCE, PMB/GCE, and PMB-ERGO/GCE.

SEM images of GCE, PMB/GCE, and PMB-ERGO/GCE are displayed in Figure 3. PMB film is smooth, dense, and uniform and PMB-ERGO film shows a rough surface feature with bulges, which indicates that ERGO is embedded into the polymer structure of MB and increases the specific surface area. Compared with bare GCE, the electropolymerized films of PMB and PMB-ERGO were colorful and could be observed by naked eye, suggesting the films had been successfully adhered to the electrode surface.

**Figure 4:**
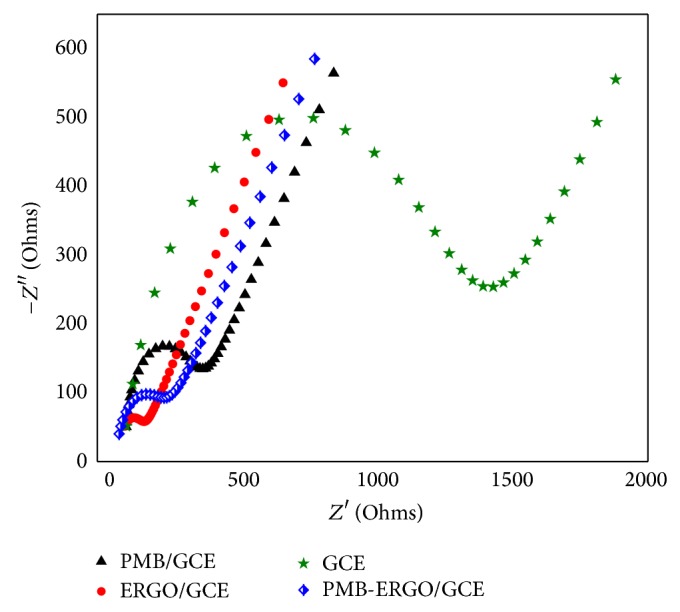
EIS of GCE, PMB/GCE, ERGO/GCE, and PMB-ERGO/GCE in 5.0 × 10^−3^ M K_3_[Fe(CN)_6_], 1.0 M KCl solution with initial *E* for 150 mV, high frequency for 2000 Hz, low frequency for 0.05 Hz, and A.C. amplitude for 150 mV.

Figure 4 depicts the electrochemical impedance spectra of GCE, PMB/GCE, ERGO/GCE, and PMB-ERGO/GCE, and their corresponding impedance values were 1212 Ω, 233.1 Ω, 96.4 Ω, and 162.5 Ω, respectively. These data imply that the modified films of PMB, ERGO, and PMB-ERGO had accelerated the rate and lowered the activation energy of electron transfer, particularly ERGO.

### 3.2. Electrocatalytic Activity of UA and Xa at Different Electrodes

**Figure 5:**
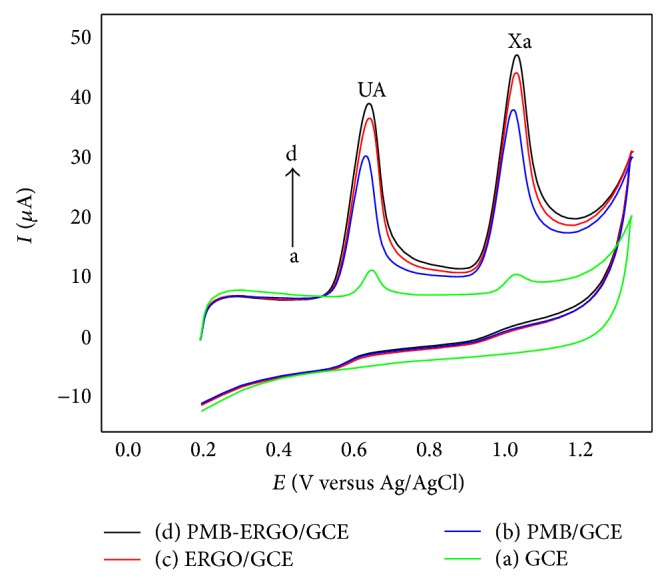
CVs of bare GCE, PMB/GCE, ERGO/GCE, and PMB-ERGO/GCE in the mixture solution of UA and Xa and PBS (pH 3.0) at a scan rate of 0.14 V*·*s^−1^ with quiet time for 2 min.

Figure 5 shows the CV responses of the bare GCE, PMB/GCE, ERGO/GCE, and PMB-ERGO/GCE toward 2.00 × 10^−4^ M UA and 2.00 × 10^−4^ M Xa.  Table 1 lists the anodic peak currents and potentials of different electrodes. It is noticed that the anodic peak potentials on the four electrodes are close. However, the anodic peak currents of UA and Xa on PMB-ERGO/GCE are much higher than others, indicating PMB-ERGO/GCE has the greatest electrochemical activity, which ought to be attributed to the synergistic effects of PMB and ERGO. On PMB-ERGO/GCE, Epa(UA) = 0.630 V, and Epa(Xa) = 1.006 V, their peaks were separated by 0.376 V. This result indicates that the simultaneous determination for UA and Xa could be achieved without separation. Combining the nice conductivity, PMB-ERGO/GCE is appropriate to be employed to investigate the electrochemical behavior of UA and Xa.

### 3.3. Effective Area of Electrodes

GCE, PMB/GCE, ERGO/GCE, and PMB-ERGO/GCE were immerged into the solution containing 5.0 × 10^−3^ M K_3_[Fe(CN)_6_] and 1.0 M KCl, followed by sweeping with cyclic voltammetry. The effective area of electrode can be calculated from the Randles-Sevcik plot: *Ip* = 2.69 × 10^5^ *n* ^3/2^ *AD* ^1/2^ *v* ^1/2^ *C*, wherein* i*_*p*_ is current in amps (A),* n* is number of electrons transferred of K_3_[Fe(CN)_6_] in the redox event (usually is 1),* A* is electrode effective area,* D* is diffusion coefficient (7.6 × 10^−6^ cm^2^ *·*s^−1^),* C* is concentration (5 mM), and *ν* is scan rate (0.05 V*·*s^−1^). The effective areas for GCE, PMB/GCE, ERGO/GCE, and PMB-ERGO/GCE were 0.0941 cm^2^, 0.1906 cm^2^, 0.2088 cm^2^, and 0.2291 cm^2^, respectively. PMB-ERGO/GCE contains the largest effective area, which presumably accounts for its excellent electrocatalytic activity.

### 3.4. Effect of pH

**Figure 6:**
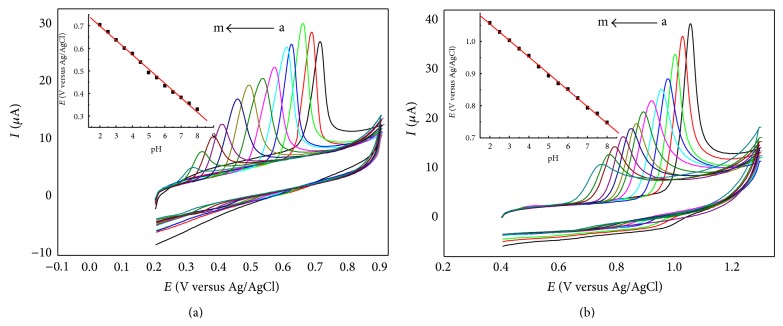
CVs of UA (a) and Xa (b) with different pH. Insets show the calibration plots of* E-pH*. From a to m: pH = 2.0, 2.5, 3.0, 3.5, 4.0, 4.5, 5.0, 5.5, 6.0, 6.5, 7.0, 7.5, 8.0.

In most cases, the electrolyte pH is an important condition to the electrochemical reaction. The cyclic voltammograms of 5.00 × 10^−5^ M UA and 1.00 × 10^−4^ M Xa were recorded from pH 2.0 to 8.0. As shown in Figure 6, the anodic peak potentials shift in negative direction with a rising value of pH, suggesting that protons have participated in electrode reactions. The anodic peak current of UA increases up to pH 3.0 and then decreases. However, the anodic peak current of Xa decreases in response to increasing pH.  Table 2 lists the relationship between peak potential and pH of UA and Xa. Based on the analysis above, pH 3.0 and pH 2.0 were used in individual determination for UA and Xa, respectively, and pH 3.0 was used in their simultaneous determination.

### 3.5. Effect of Scan Rates

**Figure 7:**
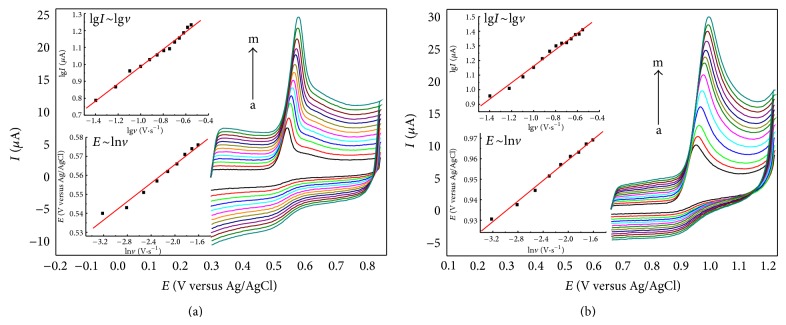
CVs for UA (a) and Xa (b) at different scan rate in PBS (pH 3.0). Insets show the calibration plots of* lgI-lgv* and* E-lnv*. From a to m: 0.04, 0.06, 0.08, 0.10, 0.12, 0.14, 0.16, 0.18, 0.20, 0.22, 0.24, 0.26, 0.28.

To investigate the effect of scan rate on the electrochemical behavior of UA and Xa (2.50 × 10^−5^ M) on PMB-ERGO/GCE, cyclic voltammograms were performed by various potential scan rates.  Figure 7 shows the anodic peak potential shifts to positive region with increasing scan rates; in addition, the anodic peak current increases at the same time. The relationships between scan rate, peak current, and peak potential are expressed with equations in Table 3.

**Figure 8:**
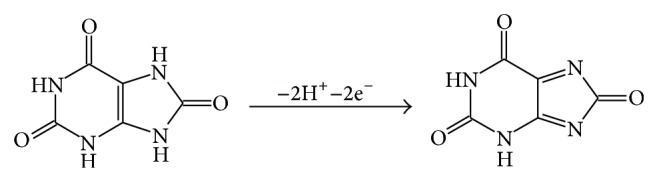
The electrochemical reaction mechanism for UA.

**Figure 9:**
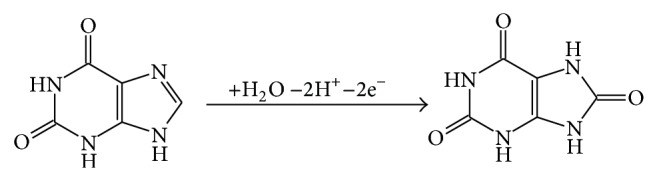
The electrochemical reaction mechanism for Xa.

The slope values approximate to 0.5 in equations of* lgI-lgv*, which clearly reveals that the electron transfer reactions of UA and Xa are both controlled by diffusion. Formula* RT/(*α*nF)* is usually used to estimate the slope in* E-lnv* relationship [36]; herein,* n* is the reaction electron number,* F* is Faraday constant,* R* is gas constant, and *α* represents electron transfer coefficient (usually is 0.5). The real reaction electron numbers of UA and Xa were calculated to be 1.964 and 1.913, respectively. Their theoretical reaction electron numbers should be 2. According to literatures [27, 37, 38], the mechanisms of UA and Xa oxidation can be proposed as follows in Figures 8 and 9, respectively.

### 3.6. Individual and Simultaneous Determination of UA and Xa

**Figure 10:**
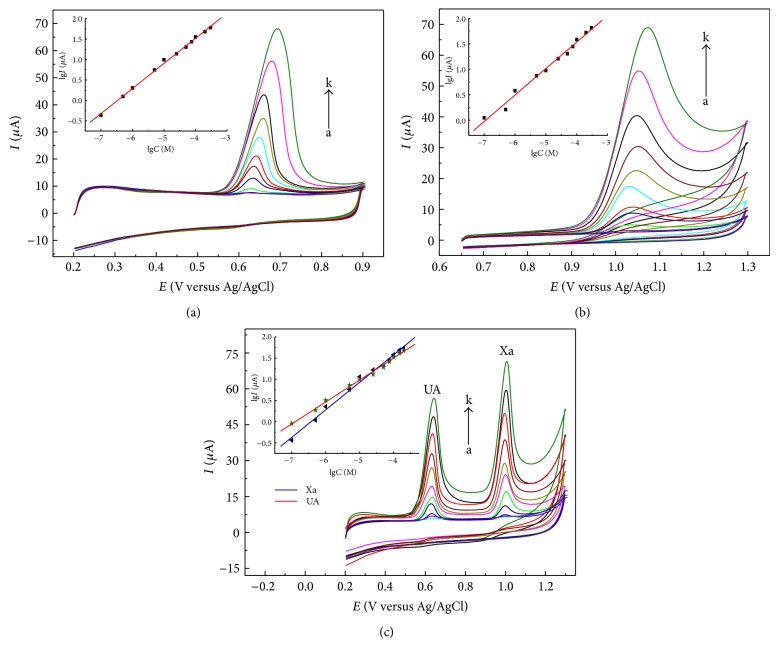
CVs of different concentrations of UA (a), Xa (b), and the mixture solution of UA and Xa (c) (from a to k: 0.08, 0.1, 0.5, 1, 2.5, 5, 7.5, 10, 25, 50, 75, 100, 200, 300, 400 *μ*M) at a scan rate of 0.14 V*·*s^−1^ with quiet time for 2 min. Insets show the calibration plots of* lgI-lgC*.

**Figure 11:**
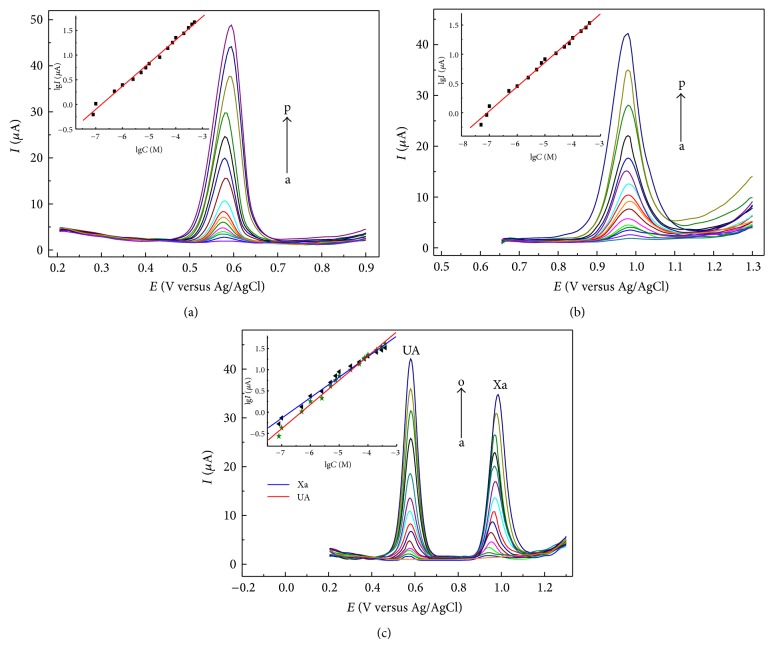
SWVs of different concentrations of UA (a) (from a to p: 0.08, 0.1, 0.5, 1, 2.5, 5, 7.5, 10, 25, 50, 75, 100, 200, 300, 400, 500 *μ*M), Xa (b) (from a to p: 0.05, 0.08, 0.1, 0.5, 1, 2.5, 5, 7.5, 10, 25, 50, 75, 100, 200, 300, 400 *μ*M), and the mixture solution of UA and Xa (c) (from a to o: 0.08, 0.1, 0.5, 1, 2.5, 5, 7.5, 10, 25, 50, 75, 100, 200, 300, 400 *μ*M) with step *E* for 5 mV, amplitude for 30 mV, frequency for 5 Hz, and quiet time for 2 min. Insets show the calibration plots of* lgI*-*lgC*.

Figures 10 and 11 describe CV and SWV responses of UA, Xa, and their mixture solution at various concentrations, respectively. And Table 4 lists their linear ranges, detection limits, and so forth.

The results in Table 4 demonstrated that the proposed method had wide linear range and high sensitivity. The comparison between this method and other electrochemical methods for simultaneous determination of UA and Xa was listed in Table 5.

### 3.7. Reproducibility and Stability

The mixed solution of 5.00 × 10^−5^ M UA and 1.00 × 10^−4^ ****M Xa were SWV measured for 20 parallel experiments. The relative standard deviations (RSD) of the peak currents for UA and Xa were 3.1% and 2.7%, respectively, implying remarkable reproducibility. After the modified electrode was stored in humid environment at room temperature for 15 days, it retained 94.5% of its original response and held the similar shape of the original curves, suggesting an acceptable stability of PMB-ERGO/GCE.

### 3.8. Interference

The influences of various foreign species were investigated in a mixture solution containing 2.00 × 10^−5^ ****M UA and 5.00 × 10^−5^ ****M Xa. The tolerance limit was set as the maximum concentration of the foreign substances that caused an approximately ±5% relative error in the determination. The results showed that K^+^, Na^+^, Ca^2+^, Fe^2+^, Mg^2+^, Ba^2+^, Zn^2+^, Al^3+^, Cl^−^, NO_3_^−^, SO_4_^2−^, C_2_O_4_^2−^, starch, L-arginine, L-threonine, L-serine, L-histidine, dopamine (≥1.0** **mg) and L-cysteine (0.6** **mg), Cu^2+^ (0.6** **mg), Ag^+^ (0.05** **mg), I^−^ (0.03** **mg), and ascorbic acid (0.5** **mg) had no interference with the determination of UA and Xa. Therefore, it is possible to simultaneously determine UA and Xa in a sample on PMB-ERGO/GCE.

### 3.9. Sample Analysis

Human urine sample was selected as the real sample to examine the reliability of the proposed SWV method. 1 mL urine sample was diluted 10 times with 0.1** **M PBS (pH 3.0) before measurement. The obtained results are summarized in Table 6.

## 4. Conclusions

PMB-ERGO/GCE was prepared by electropolymerization. The modified electrode exhibited good conductivity and excellent electrocatalytic activity toward UA and Xa. The introduction of ERGO enhanced the effective surface areas on modified electrode compared with PMB film. The real reaction electron number of UA and Xa were calculated to be 1.964 and 1.913, respectively, in pH 3.0 phosphate buffer solution. The oxidation of UA and Xa were both controlled by diffusion. PMB-ERGO/GCE displayed desirable properties including excellent stability, reproducibility, selectivity, and sensitivity. The results demonstrated that the proposed method is a rapid, sensitive, and reproducible method for determination of UA and Xa in human urine sample. Therefore, PMB-ERGO/GCE would act as a promising sensor for a wide range of electrochemical sensing and biosensing applications.

## Figures and Tables

**Table 1:** Detail data of cyclic voltammograms at different electrodes.

Electrode	UA		Xa	
	*E*: V	*I*: *μ*A	*E*: V	*I*: *μ*A
Bare GCE	0.638	4.542	1.004	3.569
PMB/GCE	0.622	24.53	0.998	28.79
ERGO/GCE	0.632	30.56	1.004	34.46
PMB-ERGO/GCE	0.630	33.91	1.006	36.81

**Table 2:** The relationship between peak potential and pH of UA and Xa.

Analye	Scan rate *v*: V*·*s^−1^	The range of pH	Linear regression equation *E*: V	Correlation coefficient
UA	0.14	2.0~8.0	*E* = 0.8263 − 0.06361pH	0.9942
Xa			*E* = 1.158 − 0.05156pH	0.9916

**Table 3:** The relationship between scan rate, peak current, and peak potential.

Analye	The relationship between scan rate and peak current			The relationship between scan rate and peak potential		
	Scan rate range *v*:* *V*·*s^−1^	Linear regression equation *I*:* * *μ*A *v*:* *V*·*s^−1^	Correlation coefficient	Scan rate range *v*:* *V*·*s^−1^	Linear regression equation *E*:* *V *v*:* *V*·*s^−1^	Correlation coefficient
UA	0.04~0.28	lg*I* = 1.499 + 0.5114lg*v*	0.9948	0.04~0.20	*E* = 0.6189 + 0.02615ln⁡*v*	0.9909
Xa		lg*I* = 1.724 + 0.5632lg*v*	0.9922		*E* = 1.011 + 0.02685ln⁡*v*	0.9927

**Table 4:** Analytical parameters for individual and simultaneous determination of UA and Xa at PMB-ERGO/GCE.

Measurement method	Analye	Analysis Method	Linear range * *(M)	Linear regression equation *I*:* * *μ*A *C*:* *M	Correlation coefficient	Detection limit * *(M)
Individual	UA	CV	1.00 × 10^−7^~3.00 × 10^−4^	lg*I* = 3.981 + 0.6148lg*C*	0.9958	8.00 × 10^−8^
		SWV	8.00 × 10^−8^~5.00 × 10^−4^	lg*I* = 3.187 + 0.4697lg*C*	0.9933	3.00 × 10^−8^
	Xa	CV	1.00 × 10^−7^~3.00 × 10^−4^	lg*I* = 3.615 + 0.5201lg*C*	0.9942	8.00 × 10^−8^
		SWV	5.00 × 10^−8^~4.00 × 10^−4^	lg*I* = 2.937 + 0.4155lg*C*	0.9966	1.00 × 10^−8^

Simultaneous	UA	CV	1.00 × 10^−7^~2.00 × 10^−4^	lg*I* = 3.572 + 0.5180lg*C*	0.9926	8.00 × 10^−8^
		SWV	8.00 × 10^−8^~4.00 × 10^−4^	lg*I* = 3.604 + 0.5702lg*C*	0.9940	3.00 × 10^−8^
	Xa	CV	5.00 × 10^−7^~3.00 × 10^−4^	lg*I* = 4.179 + 0.6501lg*C*	0.9931	1.00 × 10^−7^
		SWV	1.00 × 10^−7^~4.00 × 10^−4^	lg*I* = 3.240 + 0.4832lg*C*	0.9937	5.00 × 10^−8^

**Table 5:** Comparison of the proposed method with other electrochemical methods for the simultaneous determination of UA and Xa.

Electrode	Linear range (M)	Detection limit (M)	Reference
Poly (ATD) modified glassy carbon electrode	UA: 5.0 × 10^−6^ ~ 4.5 × 10^−5^ Xa: 5.0 × 10^−6^ ~ 4.5 × 10^−5^	UA: 1.9 × 10^−7^ Xa: 5.9 × 10^−7^	[30]

Poly (BCP) modified glassy carbon electrode	UA: 5.0 × 10^−7^ ~ 1.2 × 10^−4^ Xa: 1.0 × 10^−7^ ~ 1.0 × 10^−4^	UA: 2.0 × 10^−7^ Xa: 6.0 × 10^−8^	[31]

Electrochemically reduced graphene oxide modified electrode	UA: 5.0 × 10^−7^ ~ 6.0 × 10^−5^	UA: 5.0 × 10^−7^	[32]

Preanodized nontronite coated screen-printed carbon electrode	UA: 2.0 × 10^−6^ ~ 4.0 × 10^−5^ Xa: 2.0 × 10^−6^ ~ 4.0 × 10^−5^	UA: 4.2 × 10^−7^ Xa: 7.0 × 10^−8^	[33]

Poly (L-arginine)/graphene composite film modified electrode	UA: 1.0 × 10^−7^ ~ 1.0 × 10^−5^ Xa: 1.0 × 10^−7^ ~ 1.0 × 10^−5^	UA: 5.00 × 10^−8^ Xa: 5.00 × 10^−8^	[34]

Poly(methylene blue) and electrochemically reduced graphene oxide composite film modified electrode	UA: 8.00 × 10^−8^ ~ 4.00 × 10^−4^ Xa: 1.00 × 10^−7^ ~ 4.00 × 10^−4^	UA: 3.00 × 10^−8^ Xa: 5.00 × 10^−8^	This work

**Table 6:** Simultaneous determination of UA and Xa in human urine samples (*n* = 5).

Analye	Original value (M)	Average (M)	RSD (%)	Added (M)	Recovery (%)
UA	1.46 × 10^−5^ 1.32 × 10^−5^ 1.44 × 10^−5^ 1.37 × 10^−5^ 1.42 × 10^−5^	1.41 × 10^−5^	4.24	1.50 × 10^−5^	97.8

Xa	1.87 × 10^−7^ 1.77 × 10^−7^ 1.76 × 10^−7^ 1.91 × 10^−7^ 1.78 × 10^−7^	1.82 × 10^−7^	3.29	1.50 × 10^−7^	106
